# Synchronous Langat Virus Infection of *Haemaphysalis longicornis* Using Anal Pore Microinjection

**DOI:** 10.3390/v9070189

**Published:** 2017-07-17

**Authors:** Melbourne Rio Talactac, Kentaro Yoshii, Emmanuel Pacia Hernandez, Kodai Kusakisako, Remil Linggatong Galay, Kozo Fujisaki, Masami Mochizuki, Tetsuya Tanaka

**Affiliations:** 1 Laboratory of Infectious Diseases, Joint Faculty of Veterinary Medicine, Kagoshima University, 1-21-24 Korimoto, Kagoshima 890-0065, Japan; talactacdvm@cvsu.edu.ph (M.R.T.); jhunemman@yahoo.com (E.P.H.); seigi26jp@yahoo.co.jp (K.K.); masamimochizuki@gmail.com (M.M.); 2 Department of Pathological and Preventive Veterinary Science, The United Graduate School of Veterinary Science, Yamaguchi University, Yoshida, Yamaguchi 753-8515, Japan; 3 Laboratory of Public Health, Faculty of Veterinary Medicine, Hokkaido University, Kita-ku Kita-18 Nishi-9, Sapporo, Hokkaido 060-0818, Japan; kyoshii@vetmed.hokudai.ac.jp; 4 Department of Veterinary Paraclinical Sciences, College of Veterinary Medicine, University of the Philippines Los Baños, Los Baños, Laguna 4031, Philippines; rlgalay.dvm@gmail.com; 5 Department of Clinical and Population Health, College of Veterinary Medicine and Biomedical Sciences, Cavite State University, Cavite 4122, Philippines; 6 National Agriculture and Food Research Organization, 3-1-5 Kannondai, Tsukuba, Ibaraki 305-0856, Japan; acarikf@nifty.com

**Keywords:** anal pore microinjection, Langat virus, *Haemaphysalis longicornis*, virus transmission

## Abstract

The tick-borne encephalitis virus (TBEV) serocomplex of flaviviruses consists of arboviruses that cause important diseases in animals and humans. The transmission of this group of viruses is commonly associated with tick species such as *Ixodes* spp., *Dermacentor* spp., and *Hyalomma* spp. In the case of *Haemaphysalis longicornis*, the detection and isolation of flaviviruses have been previously reported. However, studies showing survival dynamics of any tick-borne flavivirus in *H. longicornis* are still lacking. In this study, an anal pore microinjection method was used to infect adult *H. longicornis* with Langat virus (LGTV), a naturally attenuated member of the TBEV serocomplex. LGTV detection in ticks was done by real-time PCR, virus isolation, and indirect immunofluorescent antibody test. The maximum viral titer was recorded at 28 days post-inoculation, and midgut cells were shown to be the primary replication site. The tick can also harbor the virus for at least 120 days and can successfully transmit LGTV to susceptible mice as confirmed by detection of LGTV antibodies. However, no transovarial transmission was observed from the egg and larval samples. Taken together, our results highly suggest that anal pore microinjection can be an effective method in infecting adult *H. longicornis*, which can greatly assist in our efforts to study tick and virus interactions.

## 1. Introduction

Ticks are important vectors of disease-causing pathogens in domestic and wild animals and are considered to be second to mosquitoes in transmitting human diseases [1]. Among the important pathogens that ticks transmit, viruses remain a big threat to both human and animal populations, as they can produce diseases with high morbidity and mortality [2]. Flaviviruses account for the majority of arthropod-borne viruses worldwide, including the tick-borne encephalitis virus (TBEV) serocomplex [3,4]. TBEV serocomplex consists of arboviruses that cause important diseases in animals and humans. The transmission of this group of viruses is commonly associated with tick species that include hard ticks *Ixodes ricinus*, *I. persulcatus*, *Dermacentor* spp., and *Hyalomma* spp. [3].

*Haemaphysalis longicornis*, a hard tick mainly distributed in East Asia and Australia, is also a known vector of theileriosis and non-zoonotic babesiosis [5,6]. *H. longicornis* was also recently established as a potential reservoir and vector of a bunyavirus, the severe fever with thrombocytopenia syndrome virus (SFTSV) [7]. The detection and isolation of flaviviruses have been reported previously in *H. longicornis* [8,9], but based on our knowledge, studies showing the survival dynamics of any tick-borne flavivirus in *H. longicornis* are still lacking. While ticks can be naturally infected with tick-borne viruses by feeding them on viraemic animals, this method requires a sufficient level of viraemia for transmission to a naïve tick [10]. Moreover, synchronization of tick infection with a defined viral inoculum is a notable limitation in this method [2]. Another method of tick infection is through percoxal microinjection, however, it bypasses the midgut barrier which makes it non-representative of natural route of infection and may not ensure consistent infection rates among fed ticks [11]. Immersion method, on the other hand, can also successfully infect ticks. This infection method is simpler and relatively inexpensive; however, generating cohorts of infected ticks with equal pathogen burden is its major limitation [12]. 

In this study, we have demonstrated a consistent infection and maintenance of Langat virus (LGTV), a naturally attenuated member of the TBEV serocomplex of flaviviruses, in adult *H. longicornis* using anal pore microinjection originally used to infect ticks with *Borrelia burgdorferi* [12]. Although no transovarial transmission was observed in this study, the ticks infected by this method successfully established horizontal transmission of LGTV to mice making this method an additional tool in studying tick–virus–host interactions. 

## 2. Materials and Methods

### 2.1. Ticks and Animals

Parthenogenetic *H. longicornis* (Okayama strain) ticks were maintained for several generations by feeding on the ears of Japanese white rabbits (KBT Oriental Co., Saga, Japan) at the Experimental Animal Center, Joint Faculty of Veterinary Medicine, Kagoshima University, Kagoshima, Japan. Alternatively, ticks were capsule/tube fed using six-week-old, female, ICR mice (Kyudo, Fukuoka, Japan). Animals in our experiments were used in accordance with approved guidelines (approval numbers VM 15005 and VM 15058) from the Animal Care and Use Committee of Kagoshima University.

### 2.2. Cells and Virus

Baby hamster kidney (BHK-21) cells (ATCC CCL-10, ATCC, Manassas, VA, USA) were maintained in Eagle’s Minimum Essential Medium (EMEM) supplemented with 5% fetal bovine serum (FBS; Equitech-Bio, Kerrville, TX, USA) and 1% antibiotic/antimycotic (Nacalai Tesque, Kyoto, Japan). Cell cultures were maintained at 37 °C under 5% CO_2_ until use. To amplify the LGTV TP21 strain, BHK-21 cells were utilized in this study. The LGTV stock titer was determined through focus formation assay, as described previously [13], and later aliquoted and stored at −80 °C.

### 2.3. Tick Infection

Adult ticks were infected with LGTV by anal pore microinjection [12], basically as described. Briefly, several 10 µL calibrated capillary tubes (Drummond Scientific Co., Broomall, PA, USA) were fabricated into microinjection needles by heating and pulling in a capillary pipette puller (model PN-30) (Narishige, Tokyo, Japan), which were eventually stored on adhesive tape in a petri dish. The ticks were then immobilized using a double-sided adhesive tape on glass slides, with the tick’s ventral side up. Under a dissecting microscope (Olympus, Tokyo, Japan), the tick’s anal aperture area was focused. Then, after connecting the microinjection needle in the IM 300 microinjector (Narishige, Tokyo, Japan) equipped with automated foot control, the tip of the tube was snapped where the diameter is slightly smaller than that of the anal aperture of the tick by gently touching the tip of the needle. Then microinjection needle was loaded with 0.3 µL of virus stock containing approximately 15,000 focus forming units (ffu) of LGTV. With the immobilized ticks under the dissecting microscope and focused on the anal aperture, a very mild pressure was gently applied to any area near the anal aperture using fine forceps, allowing the separation of the anal plates and opening the anal pore. The tip of the needle was then carefully inserted slightly into the anal aperture through the forced opening of the anal plates, while keeping the needle insertion to a minimum to prevent any damage to the hindgut. Then using the microinjector, the virus inoculum was injected to each tick, wherein, each tick received a single injection. For the control group, EMEM was injected. After the injection, the ticks were held for 24 h in a 25 °C incubator to check for any mortality arising from possible injury due to the injection.

### 2.4. Detection of Langat Virus RNA

To determine viral infection, ticks and mouse samples were collected at indicated time points and subsequently homogenized to isolate the total RNA for cDNA synthesis. Real-time PCR using THUNDERBIRD SYBR qPCR Mix (Toyobo, Osaka, Japan) with a 7300 real-time PCR system (Applied Biosystems, Foster City, CA, USA) was used to detect the viral RNA. LGTV membrane associated glycoprotein precursor (*Pre-M*) gene-specific primers were used to detect LGTV RNA, while mouse *β-actin*-specific primers were used for normalization (Table 1). Likewise, to quantify the change of viral RNA in ticks post-infection, real-time PCR was also used. *LGTV* negative-sense RNA-specific primers described elsewhere [2] were used to detect LGTV RNA, while *H. longicornis ribosomal protein L23* gene-specific primers were used for normalization (Table 1).

Alternatively, viral infection in different stages of ticks (egg, larva, and adult) was also determined using reverse transcription PCR (RT-PCR). Homogenization of tick samples, cDNA synthesis, and PCR product visualization were already described elsewhere [14]. Detection of viral RNA was carried out using universal flavivirus forward (5′-AATGTACGCTGATGACACAGCTGGCTGGGACAC-3′) and reverse (5′-TCCAGACCTTCAGC ATGTCTTCTGTTGTCATCCA-3′) primers [15], while *H. longicornis actin* gene-specific forward (5′-ATCCTGCGTCTCGACTTGG-3′) and reverse (5′-GCCGTGGTGGTGAAAGAGTAG-3′) primers [16] were used as internal control.

### 2.5. Langat Virus Titration Among LGTV-Infected Adult Ticks

Ticks inoculated with LGTV were collected and individually homogenized at 0, 1, 3, 7, 14, 21, 28, 60, and 120 days post-inoculation (dpi). The collected individual homogenate was eventually titrated as previously described [13].

### 2.6. Detection of Langat Virus Antigens in Tick Organs Using Indirect Immunofluorescent Antibody Test

The indirect immunofluorescent antibody test (IFAT) was performed to demonstrate the localization of LGTV in salivary glands, midguts, and hemocytes of *H. longicornis* (28 dpi via anal pore microinjection), following the method described previously [17,18].

### 2.7. Langat Virus Transmission from Ticks to Mice

To determine whether LGTV could be transmitted to mice by tick bite, LGTV-infected adult ticks (28 dpi) were allowed to feed on 20 mice (one tick per mouse) until fully engorged by feeding capsule method, as previously described [19]. Blood samples collected from each mouse at 28 days after infestation (dai) were used for LGTV detection using specific real-time PCR primer pairs (Table 1). Then, using the previously described immunofluorescence assay (IFA) [7], we also detected LGTV antibodies in serum samples from mice fed upon by infected adult ticks. Likewise, mice were observed for up to 28 dai for any clinical signs, including hunchback posture, ruffled fur, and hind-limb paralysis [20,21]. Brain samples were collected from mice that exhibited paralysis (considered terminal) and from survivors (live mice 28 dai) to detect viral RNA, as described above. Lastly, five mice infested with EMEM-injected ticks and another five mice injected with 10,000 ffu of LGTV intraperitoneally served as negative and positive controls, respectively. 

### 2.8. Transovarial Transmission of Langat Virus in Ticks

All the fully engorged ticks collected from the tick infestation experiment using feeding capsule method were collected and allowed to lay eggs. Fifty percent of the individual egg clutch produced from both LGTV- and EMEM-injected groups was homogenized separately, while the remaining 50% of each egg clutch was allowed to hatch into larvae. Both egg and larval homogenates were used to isolate total RNA for cDNA synthesis. LGTV RNA was detected in each sample using RT-PCR.

### 2.9. Statistical Analysis

All samples were tested at least in triplicate and statistically analyzed using Welch’s *t*-test in GraphPad Prism version 3.0 software (GraphPad Software, San Diego, CA, USA), wherein *p*-values of less than 0.05 and 0.01 were regarded as significant and highly significant, respectively. 

## 3. Results

### 3.1. Langat Virus Infection in Ticks

We initially observed that LGTV injected ticks via anal pore microinjection remained positive to LGTV RNA even after 28 dpi (Table 2).

Additionally, infectious virions can be detected in the midgut (20/20) and carcass (without the midgut and salivary gland; 3/20), but not in the salivary gland (0/20) (Table 3).

**Figure 1:**
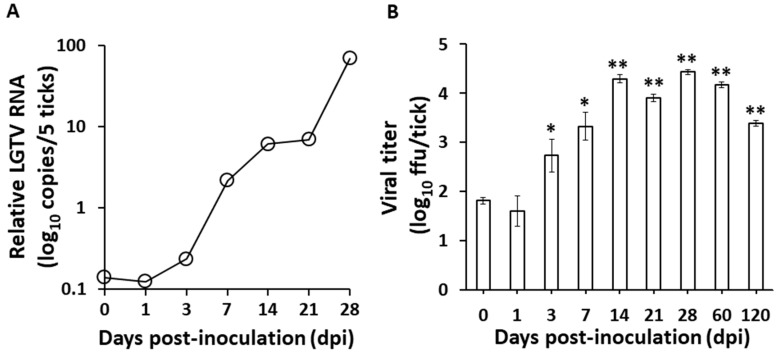
Replication of Langat virus in *Haemaphysalis longicornis* after infection via anal pore microinjection. (**A**) Real-time PCR was used to quantify the changes in the negative-sense strand of LGTV RNA collected from groups of five ticks at each time point. The *H. longicornis L23* gene was used to normalize the data at each time point. (**B**) Virus titration after LGTV infection via anal pore microinjection. Error bars in virus titers indicate the SD in mean values of three ticks at each time point. * *p* < 0.05, ** *p* < 0.01, as compared to day 0.

These observations may suggest that the virus can be maintained in the ticks, specifically in the midgut, even after 28 dpi without any difference in mortality compared to the control. However, since the mere presence of infectious virions may only suggest retention and not necessarily replication, we later on confirmed the LGTV replication through time point determination of LGTV RNA and titer among the anal pore microinjected ticks. The LGTV negative-sense RNA strand, an obligatory marker for virus replication [2], increased over the time course of the infection (Figure 1A), and increasing viral titers were also observed beginning 3 dpi, with the maximum titer recorded at 28 dpi, while the virus remained detectable for at least 120 dpi (Figure 1B).

**Figure 2:**
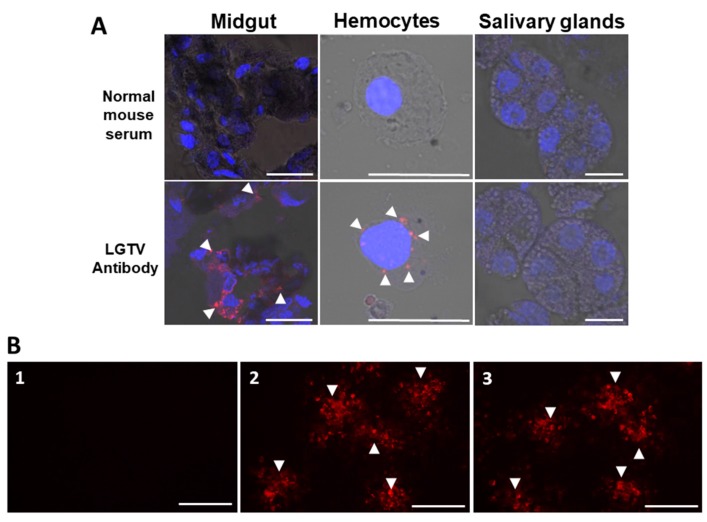
Localization of Langat virusin selected organs from unfed adult ticks after infection via anal pore microinjection and immunofluorescence assay detection of LGTV antibodies in serum samples from mice. (**A**) Viral antigens were detected using a specific LGTV polyclonal antibody, while normal mouse serum served as a control. Nuclei counterstaining (blue) was done using DAPI, and arrowheads denote LGTV antigens (red) (bar = 20 μm. (**B**) Sera collected from mouse infested with EMEM-injected tick (**1**) (1:200, No.1); mouse inoculated with 10,000 ffu of LGTV (**2**) (1:12,800, No.1); mouse infested with LGTV-injected tick (**3**) (1:6,400, No.18) reacting with LGTV-infected baby hamster kidney cells. Arrowheads denote LGTV ffu detected by LGTV antibodies (red) (bar = 10 μm).

On the other hand, viral antigens at 28 dpi were consistently detected in the cytoplasm of midgut cells of LGTV-injected ticks (Figure 2A). We also detected LGTV antigens in the hemocytes, but not in the salivary glands.

### 3.2. Langat Virus Transmission to Mice

We also tested whether anal pore microinjected, unfed, adult ticks can successfully transmit the virus to a susceptible host. Infected adult ticks (28 dpi) were allowed to feed on mice, and then the presence of both viral RNA and LGTV antibodies was checked 28 dai. Mice were also observed for clinical signs for the entire duration of the study, wherein paralyzed mice were sacrificed to collect the brains for LGTV RNA detection through real-time PCR. Mice inoculated with LGTV or EMEM through intraperitoneal inoculation served as positive and negative controls, respectively. One mouse from the positive control group and two mice infested with the LGTV-infected ticks that showed hind-limb paralysis were positive for viral RNA in the brain (Table 4). 

In the blood and brain samples collected from the surviving mice at 28 dai, no LGTV RNA was detected; however, 16 of 18 mice (88.8%) infested with LGTV-infected ticks and 4/4 (100%) of the positive control mice showed LGTV-specific antibodies. As expected, no LGTV-specific antibodies were detected from the EMEM-inoculated ticks (Table 4). Representative IFA detection images of LGTV antibodies from all experimental groups are shown in Figure 2B.

### 3.3. Transovarial Transmission of Langat Virus in Ticks

To observe the vertical transmission of LGTV in ticks, eggs, and larvae from the infected adults were examined for viral RNA using RT-PCR. In summary, 85% (17/20) and 100% (5/5) of the engorged ticks from the LGTV- and EMEM-injected groups successfully laid eggs, respectively. In addition, a 100% hatching rate was observed in both groups. However, as shown in Table 5, no LGTV RNA was detected in both egg (0/17) and larval (0/17) samples from LGTV-injected ticks. As expected, no viral RNA was detected in the control group (0/5).

## 4. Discussion

One of the most important determinants of vector competence is the susceptibility of midgut cells to virus infection [22], and anal pore microinjection method may prove to be an important technique in evaluating vector competency, since the virus will first come in contact with the tick gut. As shown in Figure 1, LGTV successfully replicated in the *H. longicornis* as shown by increasing viral RNA and titer. We also managed to clearly demonstrate that the virus successfully infected the midgut cells and hemocytes (Figure 2) and infectious virions can be consistently isolated from the midgut (Table 3). The isolation of LGTV in some tick carcasses may suggest infection of some tick organs or may be due to incomplete washing of infected hemocytes.

On the other hand, LGTV localization and isolation in the salivary glands (Figure 2 and Table 3) was not observed in the current study. We can only speculate that the virus replicated in a sub-detectable level or may have not yet entered the organ, since we have not yet checked for the presence the virus in the salivary glands during or after blood feeding. The latter observation was previously reported in Thogoto virus (THOV), wherein the virus was not detected in the salivary glands of *Rhipicephalus appendiculatus* (transstadially infected) until the ticks had fed on a host for about seven days [23]. It was previously reported for TBEV that feeding enhances salivary gland infection; thus, partially fed ticks have significantly higher infection prevalences than unfed ticks from the same collection site [24]. It is also during a subsequent meal, that the virus enters the saliva through the salivary gland epithelium for eventual transmission [25]. Thus, despite the absence of detection of LGTV in the salivary glands of the LGTV-injected ticks, the horizontal transmission of the virus was still observed as shown in Table 4.

On the other hand, despite the failure to detect viraemia in all the experimental groups of mice, almost 90% of the naïve mice infested with infected ticks seroconverted, suggesting that subclinical infection may have occurred. Such detection of antibodies in mice already suggests virus transmission, since host infection has usually been detected by virus isolation and/or seroconversion [26]. Likewise, the two paralyzed mice infested with infected ticks showed the presence of LGTV RNA in their brains, indicating further the successful transmission of virus from ticks to mice; however, we failed to determine whether meningitis or encephalitis was present in their respective brains. What is also notable in the present study was the absence of transovarial transmission as determined from the eggs and hatched larvae collected from the engorged infected adult ticks (Table 5). Although not all infected ticks successfully transfer the virus to their eggs [11], the absence of detection from eggs and larvae could be explained by previous reports that a large proportion of larvae may become infected by non-viraemic transmission when they co-feed with infected nymph or larvae [27]. Thus, we are currently evaluating the co-feeding transmission between adult infected ticks via anal pore microinjection and naïve nymphs so that we can establish if the current method of infection can mimic the natural spread virus of among tick populations.

## 5. Conclusions

In summary, *H. longicornis* can be efficiently infected with LGTV through anal pore microinjection. Moreover, infected *H. longicornis* can effectively transmit LGTV to a susceptible host, as shown by the presence of viral RNA in the brain of infected mice and the presence of LGTV antibodies in almost 90% of infested mice. However, demonstration of LGTV transmission through co-feeding between an infected adult and immature naïve ticks are needed to demonstrate the possible mechanism on how LGTV can circulate in the tick population, especially that no transovarial transmission was observed in the study using anal pore microinjection. Taken together, our results highly suggest that the anal pore microinjection method can be a useful technique in studying tick, virus, and host interactions.

## Figures and Tables

**Table 1:** List of real-time PCR primers used to detect Langat Virus RNA.

Primer Name	Primer Sequence
LGTV Pre-M Forward	GGATGGATTGTTGCCCAGGA
LGTV Pre-M Reverse	CCCAGCTCGAGAACCAATGT
LGTV Neg. Forward	GTCTCCGGTTGCAGGACTGT
LGTV Neg. Reverse	CTCGGTCAGTAGGATGGTGTTG
H. longicornis L23 Forward	CACACTCGTGTTCATCGTCC
H. longicornis L23 Reverse	ATGAGTGTGTTCACGTTGGC
Mouse β-actin Forward	TTCTTTGCAGCTCCTTCGTT
Mouse β-actin Reverse	ATGGAGGGGAATACAGCCC

**Table 2:** Detection of Langat virus RNA from ticks injected with LGTV and Eagle’s Minimum Essential Medium via anal pore microinjection using reverse transcription PCR.

Inoculum	LGTV Detection	
	Absolute Value	Percentage
EMEM	0/20	0
LGTV	20/20	100%

**Table 3:** Comparative Langat virus titers from selected organs of unfed adult *Haemaphysalis longicornis* at 28 days post infection (dpi).

Tissue	No. Positive/Total (%)	Mean Titer ± Standard Deviation (log_10_ ffu/Tick)
Midgut	20/20 (100)	3.37 ± 0.16
Salivary Gland	0/20 (0)	-
Carcass	3/20 (15)	2.53 ± 0.85

**Table 4:** Langat virus transmission from *Haemaphysalis longicornis* to mice.

Treatment	Moribund Mice ^a^		Survivors		
	Mortality (%) (Death/Total)	Viral RNA in Brain ^b^	Seroconversion (%) (Positive/Total)	Viral RNA in Brain ^b^	Viral RNA in Blood ^b^
EMEM-injected ticks	0 (0/5)	N.A.	0 (0/5)	−	−
LGTV-injected ticks	10 (2/20)	+ (2/2)	88.8 (16/18)	−	−
LGTV inoculated mice	20 (1/5)	+ (1/1)	100 (4/4)	−	−

^a^ Mice were considered terminal and later on sacrificed at the first signs of disease. ^b^ Real-time PCR was used for detection of LGTV in mouse tissues as represented by presence (+), absence (−) or not applicable (N.A.)

**Table 5:** Detection of Langat virus RNA in eggs and larvae using reverse transcription PCR.

Sample	Group	LGTV Detection	
		Absolute Value	Percentage
Eggs	EMEM	0/5	0
	LGTV	0/17	0
Larvae	EMEM	0/5	0
	LGTV	0/17	0
